# Non-invasive assessment of coronary artery distensibility by 3.0 T cardiac MRI

**DOI:** 10.1186/1532-429X-11-S1-P38

**Published:** 2009-01-28

**Authors:** Sebastian Kelle, Allison G Hays, Glenn A Hirsch, Gary Gerstenblith, Robert G Weiss, Matthias Stuber

**Affiliations:** Johns Hopkins University, Baltimore, MD USA

**Keywords:** Right Coronary Artery, Cardiac Magnetic Resonance Imaging, Coronary Artery Disease Patient, Aortic Distensibility, Documented Coronary Artery Disease

## Introduction

Atherosclerotic changes in the coronary artery are associated with impaired coronary vessel wall distensibility [1]. Though non-invasive measures of central aortic distensibility are possible, only intravascular ultrasound (IVUS) invasive measurements of coronary distensibility have been reported until now [1, 2].

## Purpose

High-field coronary magnetic resonance imaging (MRI) offers high temporal and spatial resolution, important for assessing distensibility-related changes in coronary dimensions during the cardiac cycle. We sought to test the hypothesis that coronary artery distensibility can be evaluated non-invasively with 3.0 T cardiac MRI and that distensibility differences can be detected between healthy (control group) and coronary artery disease (CAD) subjects.

## Methods

Twenty healthy, adult subjects (11 male, age 19–60 years, mean 32 ± 12 years) and twelve patients with coronary catheterization documented CAD (8 male, age 50–69 years, mean 58 ± 8 years) were studied on a commercial whole-body MR imaging system (Achieva 3.0 T; Philips, Best, The Netherlands). MR angiography of the right coronary artery (RCA) was performed with a navigator-gated free-breathing and ECG-triggered, T2-prepared, three-dimensional, segmented k-space, gradient-echo imaging sequence. In each subject, the proximal segment of the RCA was then imaged in cross-section using cine spiral MRI for area measurements. Imaging was performed at a constant room temperature and after at least 20 minutes of rest in the magnet. MRI parameters were: echo time (TE) = 1.5 ms, radiofrequency (RF) excitation angle = 20° and spectral spatial excitation, breath-hold duration ~14–24 sec, acquisition window = 10 ms, repetition time (TR) = 14 ms, 21 spiral interleaves, spatial resolution (acquired/reconstructed) = 0.89 × 0.89 × 8.00 mm^3^/0.69 × 0.69 × 8.00 mm^3^. Both the blood pressure and the heart rate were recorded. Images were analyzed for cross-sectional area changes using full width half maximum (Cine version 3.15.17, General Electric, Milwaukee, WI, USA), and distensibility (mmHg^-1^) was determined as: [(systolic lumen area – diastolic lumen area)]/(pulse pressure multiplied with the diastolic lumen area) [3]. Pulse pressure was calculated as pressure change during a cardiac cycle [2].

## Results

**Figure 1:**
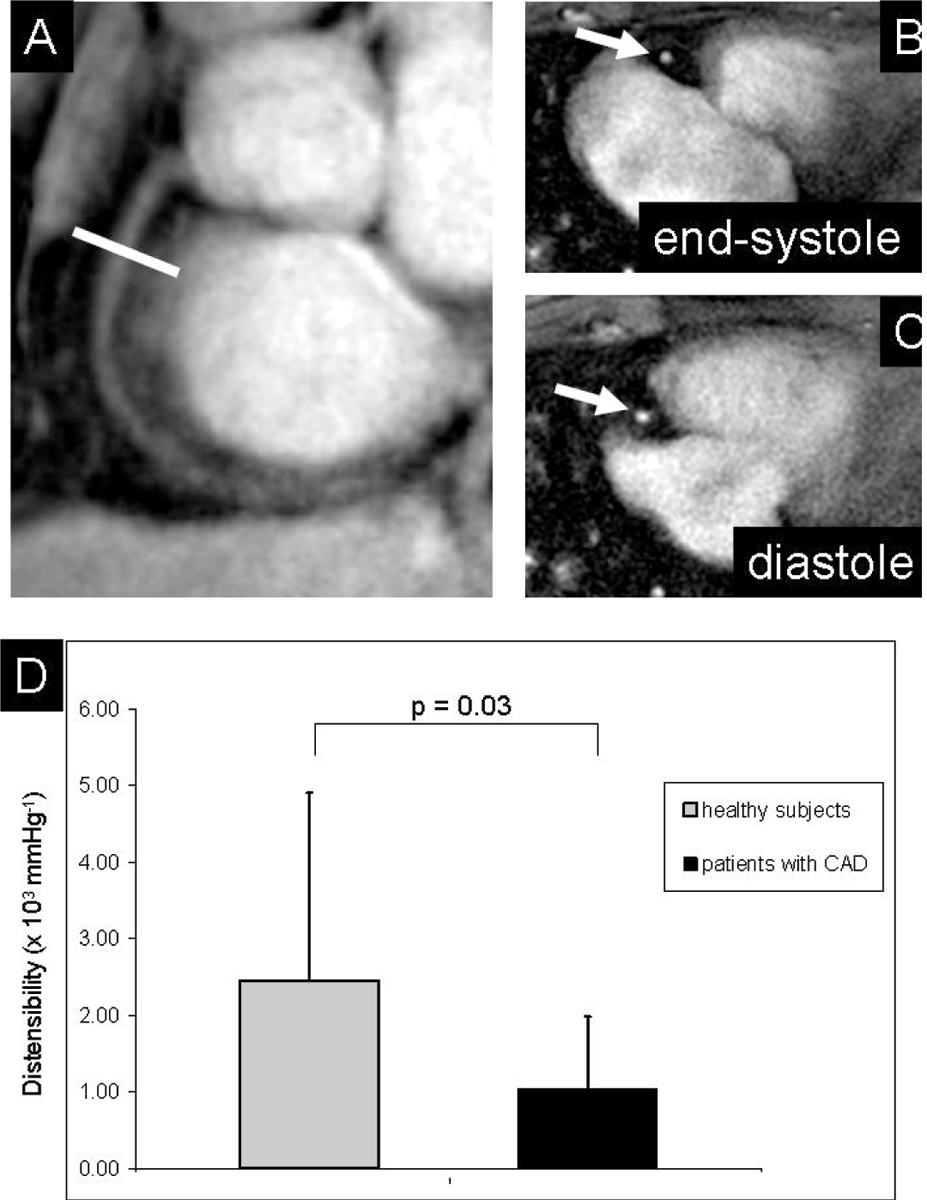
**(A): The MRA of the right coronary artery (RCA) is used to select a cross-sectional plane (indicated by white line)**. Images of the proximal RCA (white arrow) in end-systole **(B)** and at diastole **(C)** in a patient with coronary artery disease (CAD). **(D)**: Distensibility measurements in healthy subjects (N = 19) and patients with CAD (N = 11).

Nineteen volunteers and eleven patients had adequate image quality for RCA area measurements (figure 1A). The mean heart-rate pressure product (heart-rate multiplied by systolic blood pressure) in healthy adults (8289 ± 1427 mmHg*beats/minute) was not significantly different from that in CAD patients (9048 ± 1878 mmHg*beats/minute), (p = 0.18). The luminal area in healthy subjects was 9.60 ± 2.02 mm^2^ during diastole and 12.90 ± 4.78 mm^2^ for CAD patients (p = 0.01). At end-systole, the luminal area in healthy subjects was 10.60 ± 2.47 mm^2^ and 13.55 ± 5.39 mm^2^ for CAD patients respectively (p = 0.049). Coronary vessel area changed significantly between systole and diastole in healthy controls (p < 0.01), but not in CAD patients (p = 0.12). In healthy subjects coronary artery distensibility (2.46 ± 2.45 mmHg^-1^ *10^3^) was significantly higher than that in CAD patients (1.04 ± 0.94 mmHg^-1^ *10^3^) (figure 1B) (p = 0.03). We found no significant correlation between coronary artery distensibility and coronary vessel area at either systole or diastole in healthy adults or CAD patients.

## Discussion

Non-invasive assessment of coronary artery vessel wall distensibility with 3.0 T is feasible and the findings are similar to those from invasive IVUS studies [1, 2]. Coronary artery distensibility measured by 3.0 T MRI is significantly higher in healthy controls than it is in patients with documented CAD. The number of subjects needed to detect a difference is relatively small. This methodology may support the characterization of vascular anatomy and function in healthy and diseased states, as well as the response to interventions in patients with, or at increased risk for CAD.
